# Sepsis is a major determinant of outcome in critically ill HIV/AIDS patients

**DOI:** 10.1186/cc9221

**Published:** 2010-08-10

**Authors:** André M Japiassú, Rodrigo T Amâncio, Emerson C Mesquita, Denise M Medeiros, Helena B Bernal, Estevão P Nunes, Paula M Luz, Beatriz Grinsztejn, Fernando A Bozza

**Affiliations:** 1 Intensive Care Unit, Instituto de Pesquisa Clínica Evandro Chagas, Fundação Oswaldo Cruz, Av Brasil 4365, Rio de Janeiro, RJ, 21040-360, Brazil; 2 HIV/AIDS Research Centre, Instituto de Pesquisa Clínica Evandro Chagas, Fundação Oswaldo Cruz, Av Brasil 4365, Rio de Janeiro, RJ, 21040-360, Brazil

## Abstract

**Introduction:**

New challenges have arisen for the management of critically ill HIV/AIDS patients. Severe sepsis has emerged as a common cause of intensive care unit (ICU) admission for those living with HIV/AIDS. Contrastingly, HIV/AIDS patients have been systematically excluded from sepsis studies, limiting the understanding of the impact of sepsis in this population. We prospectively followed up critically ill HIV/AIDS patients to evaluate the main risk factors for hospital mortality and the impact of severe sepsis on the short- and long-term survival.

**Methods:**

All consecutive HIV-infected patients admitted to the ICU of an infectious diseases research center, from June 2006 to May 2008, were included. Severity of illness, time since AIDS diagnosis, CD4 cell count, antiretroviral treatment, incidence of severe sepsis, and organ dysfunctions were registered. The 28-day, hospital, and 6-month outcomes were obtained for all patients. Cox proportional hazards regression analysis measured the effect of potential factors on 28-day and 6-month mortality.

**Results:**

During the 2-year study period, 88 HIV/AIDS critically ill patients were admitted to the ICU. Seventy percent of patients had opportunist infections, median CD4 count was 75 cells/mm^3^, and 45% were receiving antiretroviral therapy. Location on a ward before ICU admission, cardiovascular and respiratory dysfunctions on the first day after admission, and the presence of severe sepsis/septic shock were associated with reduced 28-day and 6-month survival on a univariate analysis. After a multivariate analysis, severe sepsis determined the highest hazard ratio (HR) for 28-day (adjusted HR, 3.13; 95% CI, 1.21-8.07) and 6-month (adjusted HR, 3.35; 95% CI, 1.42-7.86) mortality. Severe sepsis occurred in 44 (50%) patients, mainly because of lower respiratory tract infections. The survival of septic and nonseptic patients was significantly different at 28-day and 6-month follow-up times (log-rank and Peto test, *P *< 0.001).

**Conclusions:**

Severe sepsis has emerged as a major cause of admission and mortality for hospitalized HIV/AIDS patients, significantly affecting short- and longer-term survival of critically ill HIV/AIDS patients.

## Introduction

The long-term survival of patients with human immunodeficiency virus (HIV) has markedly improved since the introduction of highly active antiretroviral therapy (HAART). UNAIDS/WHO estimated at 33.4 million the number of people living with HIV in December 2008 [1]. It is expected that this number will continue to grow, in particular in third-world urban centers. Recent studies have analyzed HIV/AIDS critically ill patients' characteristics, especially comparing pre- and post-HAART eras, with emphasis on causes of admission and risk factors for mortality [2-7]. A large cohort of HIV critically ill patients showed that sepsis is among the causes of ICU admissions with increasing incidence, contrary to the decreasing trend observed for acute respiratory insufficiency and *Pneumocystis jiroveci *pneumonia [8]. Other studies have shown that bacterial infection is becoming increasingly prevalent in patients with HIV admitted to the ICU, irrespective of HAART use [9-11]. However, severe sepsis has not been systematically studied with respect to its prevalence during the ICU stay, microbiologic and organ-dysfunction characteristics, and impact on the outcome.

Epidemiologic studies have shown that a fraction of 1% to 10% of the sepsis patients are composed of individuals with HIV/AIDS, according to regional differences in the prevalence of HIV infection and ICU admission practices [12-19]. Recently, analysis of a large United States database of septic hospitalized patients showed that the septic HIV patients have lower rates of ICU admission compared with non-HIV groups [20].

Despite the significant increases in survival and quality of life, HIV/AIDS patients have been systematically excluded from sepsis studies, limiting the understanding of the impact of sepsis in this population. To date, few studies have assessed prospectively the determinants of survival of critically ill HIV/AIDS patients.

In this study, we prospectively followed up HIV/AIDS critically ill patients to evaluate the key factors related to outcome, with emphasis on impact of severe sepsis on the short- and long-term survival.

## Materials and methods

### Design and setting

This prospective cohort study was conducted at the ICU of the Instituto de Pesquisa Clínica Evandro Chagas (IPEC), Fundação Oswaldo Cruz, Rio de Janeiro, Brazil. IPEC has provided care to HIV/AIDS patients in Rio de Janeiro since 1986 and, currently, more than 2,000 adult patients are actively followed up at the HIV/AIDS clinic.

At our institution, only patients for whom potentially lifespan-extending treatment is available are usually considered for ICU admission. Critically ill unstable patients with indications for intensive care and monitoring and/or need for immediate procedures or interventions are eligible for ICU admission. Treatment-limitation decisions are taken when patients do not recover from acute illness despite intensive care, or when control of the baseline condition has not been achieved. These decisions are shared by the ICU team, HIV/AIDS specialist, and family members, based on local practices [21].

The study was supported by institutional funds. The institutional review board approved the study and waived the need for informed consent. The conduct of the study did not interfere with patient-management decisions.

### Participants, data collection, and definitions

All consecutive HIV-infected patients admitted to the ICU from June 2006 to May 2008 were included in this study. AIDS cases and the occurrence of AIDS-defining diseases were set by the Centers for Disease Control and Prevention (CDC) definitions, and HAART was initiated when the CD4 cell count was below 200-350 cells/mm^3 ^[22]. Time since AIDS diagnosis was calculated from AIDS onset until ICU admission. Recent AIDS onset was defined as that occurring 2 months or less before admission. The CD4 cell count was considered when obtained within 3 months of ICU admission or measured during the first week of ICU entry. HAART was defined as the regular use of at least two nucleoside reverse transcriptase inhibitors (NRTI) plus a protease inhibitor (PI) or a nonnucleoside reverse transcriptase inhibitor (NNRTI) or a PI and an NNRTI in combination [20]. As adherence is a major component on HIV treatment efficacy, it is always checked and noted on the patient's medical charts at our institution.

In case of multiple ICU admissions, only the first one was considered. ICU causes of admission were divided into acute respiratory insufficiency, sepsis, severe neurologic disturbances, heart disease, complications of solid organ or hematologic neoplasia, metabolic disturbance, and gastrointestinal complications. Readmissions were defined for patients returning to the ICU during the same hospitalization.

A standardized data-entry form was created for the collection of demographic data, main admission diagnosis, opportunistic infections, comorbidities, location before ICU admission (emergency room, ward, or other hospital), laboratory results based on computerized laboratory records review, ICU, and hospital length of stay. The following variables were collected during the first day of the ICU stay: expanded Simplified Acute Physiology Score (SAPS) II [23,24], Sequential Organ Failure Assessment (SOFA) score [25], and use of vasopressor agents. Performance Status (PS) was calculated based on patient or surrogate information [26]. During the ICU stay, the need for mechanical ventilation for more than 24 hours, use of renal-support devices, development of shock (use of vasopressor agent to maintain mean arterial pressure higher than 70 mm Hg) and acute lung injury (defined as PaO_2_/FiO_2 _ratio less than 300) were also recorded.

Sepsis definitions were based on the ACCP/SCCM Consensus Conference [27]; sepsis was present if there was a presumed or confirmed infection, associated with at least two of the following: tachycardia >90 beats/min; tachypnea >30 cycles/min (or hypocapnia <32 mm Hg); fever (>38°C) or hypothermia (<36°C); and leukocytosis (>12,000/mm^3^) or leukopenia (<4,000/mm^3^) or the presence of more than 10% immature forms. Severe sepsis was defined if any organ dysfunction, sepsis-induced hypotension, or elevated serum lactate levels were present. Septic shock was defined as sepsis with hypotension that persisted after adequate infusion of fluids and the need for vasopressor agents.

Infections were classified as community acquired or nosocomial, according to the cutoff time point 48 h of hospital admission. Definitions of lower pulmonary tract infection, bloodstream infection, and urinary tract infection were made by following the CDC definitions [28]. Appropriate biologic material was sampled from suspected sites of infection. Microbiologic data were collected and analyzed according to the local Infection Control Committee.

### Statistical analysis

Continuous variables were summarized as medians and interquartile ranges. We compared the distribution of continuous variables by using the *t *test, and of categoric variables by using the χ^2 ^test. We calculated the Kaplan-Meier survival function stratified by sepsis. The log-rank and Peto tests were used to evaluate whether the estimated survival functions were significantly different by strata. Two outcomes were of interest: mortality within 28 days and 6 months from entry to the ICU. We used Cox proportional hazards regression analysis to measure the effect of sepsis, as well as other factors, on 28-day and 6-month mortality. We also estimated the effect of sepsis while controlling for potential confounders such as age, time since AIDS diagnosis, HAART, location before ICU, and cardiovascular and respiratory dysfunctions, in a multivariate model. The CD4 cell count was not included on the multivariate model because of the heterogeneity of the period of data collection (within 3 months of ICU admission or during the first week of ICU entry). The proportionality assumption in Cox's regression was evaluated by the estimating the correlation between survival time and Shoenfeld's standardized residuals. We used the statistical software R, version 2.9 [29] for all statistical analysis.

## Results

### Patient characteristics

During the 2-year study period, 437 HIV-positive patients were admitted to the hospital, and 101 patients became critically ill. However, 13 patients were not considered for life-sustaining measures and died in the hospital ward. One hundred seven ICU admissions of 88 patients were recorded. We analyzed only the first ICU admission of each of these 88 patients; their characteristics are shown in Table 1. In brief, the median age was 40 years, with a male predominance (76%). The median CD4 cell count was 75 (interquartile range, 32 to 227) cells/mm^3^. The median time since AIDS diagnosis was 40 months. Twenty-five patients (28%) had recently been diagnosed with AIDS; 40 patients (45%) were adherent to HAART, and the remaining 27% had irregular use of HAART. The percentage of patients using the three drug classes was as follows: NRTI (97%), PI (67%), and NNRTI (35%). HAART use did not affect hospital survival (50% mortality for HAART and non-HAART subjects). Patients not using HAART were younger (37 versus 43 years; *P *= 0.03), had more opportunistic infections (83% versus 55%; *P *< 0.01), and had less time since AIDS diagnosis (3.5 versus 68.5 months; *P *< 0.01).

**Table 1:** Demographics, HIV/AIDS characteristics, and causes of admission to ICU of all patients

	Patients (*n *= 88)
Age (years)	40 (31-47)
Male gender	67 (76%)
Time since AIDS diagnosis (months)	40 (2-90)
Recent AIDS diagnosis	25 (28%)
CD4 cell count (per mm^3^)	75 (32-227)
HAART use	40 (45%)
Cause of admission	
Acute respiratory failure	26 (29%)
Coma/torpor	20 (23%)
Sepsis	18 (20%)
Decompensated heart disease	9 (10%)
Gastrointestinal diseases	6 (7%)
Metabolic disturbance	5 (6%)
High-grade lymphoma	4 (5%)

Continuous values are shown as median and interquartile interval. AIDS, acquired immunodeficiency syndrome; ICU, intensive care unit; HAART, highly active antiretroviral therapy.

Acute respiratory failure was the cause of ICU admission for 29% of the patients, caused by bacterial pneumonia (*n *= 10), *Pneumocystis jiroveci *pneumonia (*n *= 8), tuberculosis (*n *= 6), and cardiogenic pulmonary edema (*n *= 2). Severe neurologic complications were the cause of ICU admission in 20 patients (23%), and severe sepsis was present in 18 patients (20%). Other causes of ICU admission were decompensated heart disease (*n *= 9), gastrointestinal complications (*n *= 6), metabolic dysfunction secondary to renal dysfunction or lactic acidosis related to drug adverse effects (*n *= 5), and complications of high-grade non-Hodgkin lymphoma (*n *= 4) (Table 1). Sixty-two patients (70%) had at least one opportunistic infection; *P. jiroveci *pneumonia and neurotoxoplasmosis were the most frequent opportunistic infections.

### In-hospital mortality

The observed in-hospital mortality during the study period was 49%. We compared the characteristics of patients who survived until the hospital discharge with those of patients who did not. Patients with non-Hodgkin lymphoma and gastrointestinal complications had a worse outcome (mortality, 100% and 83%, respectively), whereas patients initially seen with acute respiratory failure, neurologic disorders, and sepsis had moderate mortality rates (46%, 45%, and 50%, respectively). SAPS II (54 versus 44 points; *P *< 0.001) and SOFA score on day 1 (7 versus 4 points, *P *< 0.001) were significantly higher for nonsurvivors (Table 2). Early shock (use of vasopressor agents in the first 24 h since admission) was more common in nonsurvivors (30% versus 13%; *P *< 0.05). Nonsurvivors used more life-support devices, such as mechanical ventilation (84% versus 38%; *P *< 0.01) and renal support therapy (30% versus 7%; *P *< 0.01). Severe sepsis or septic shock was observed with higher frequency for the nonsurvivor group (67% versus 33%; *P *< 0.01). Other demographic characteristics such as age, gender, admission from emergency or ward, and Performance Status were similar in the two groups. No statistical difference was noted between the two groups regarding CD4 cell count, time since AIDS diagnosis, and HAART use.

**Table 2:** Comparison of patients according to hospital survival

	Survivors (*n *= 45)	Nonsurvivors (*n *= 43)
Age (mean ± SD)	42 (35-50)	38 (29-46)
Male gender (%)	36 (80%)	31 (72%)
Location before ICU (ward, %)	24 (53%)	29 (67%)
Performance status 3 or 4	1 (0-2)	2 (1-3)
CD4 count <50 mm^3 ^(%)	20 (44%)	21 (49%)
Length of time from AIDS diagnosis	39 (2-92)	50 (2-85)
Recent (<3 months) AIDS diagnosis	13 (29%)	13 (30%)
HAART use	20 (44%)	20 (46%)
SAPS II expanded (points)	44 (37-54)	54 (46-67)^b^
SOFA D1 (points)	4 (1-7)	7 (4-10)^b^
Mechanical ventilation	17 (38%)	36 (84%)^b^
Use of vasopressors on day 1	6 (13%)	15 (30%)^a^
Renal support	3 (7%)	13 (30%)^b^
Severe sepsis/septic shock (%)	15 (33%)	29 (67%)^b^
ICU length of stay (days)	9 (5-17)	10 (4-14)
Hospital length of stay (days)	24 (12-58)	15 (10-24)^a^

AIDS, acquired immunodeficiency syndrome; ICU, Intensive Care Unit; HAART, highly active anti-retroviral Therapy; SAPS, Simplified Acute Physiology Score; SOFA, Sequential Organ Failure Assessment. Significant differences are shown as ^a^*P *< 0.05; ^b^*P *< 0.01.

### Severe sepsis in HIV/AIDS patients

Forty-four patients (50%) had severe sepsis at ICU admission or during the ICU stay. In comparison with nonsepsis patients, sepsis patients were more likely to come from wards (75% versus 45%; *P *< 0.01), spent more time in the ICU (11 versus 7 days; *P *= 0.03), were more severely ill (SAPS II, 56 versus 44 points; *P *< 0.001; had SOFA on day 1, 7 versus 3 points; *P *< 0.001), and needed mechanical ventilation more often (86% versus 34%; *P *< 0.001). We did not observe differences in CD4 cell count or time since AIDS diagnosis between septic and nonseptic groups. Hospital mortality was significantly higher in septic when compared to nonseptic patients (66% versus 34%; *P *= 0.002). Lung was the most common site of infection (52%), followed by primary bloodstream infections (38%), venous catheter-related bacteremia (7%), and urinary tract infections (3%) (Table 3). Nosocomial infections were the source of sepsis in 90% of the cases in our cohort. Microbiology of infections was mostly composed of gram-negative rods, such as *Pseudomonas aeruginosa *(10), *Klebsiella pneumoniae *(six), *Enterobacter *sp. (five), *Escherichia coli *(three), *Acinetobacter *sp. (three), *Serratia marcescens *(three), and *Staphylococcus *sp. (nine). *Mycobacterium tuberculosis *was the etiologic agent of severe sepsis in six (14%) patients. There were also single cases of *S. maltophilia*, *C. difficile*, *C. freundi*, *B. cepacia*, and *Candida *sp. Bacteremia was detected in 43% (19) of the sepsis patients, irrespectively of the site of infection.

**Table 3:** Site of infection and microbiologic data of septic shock AIDS patients

Site of infection	*n*
Pulmonary	23 (52%)
Primary bacteremia	17 (38%)
Venous catheter-related infection	3 (7%)
Urinary tract infections	1 (3%)

**Microbiology of infection**	**N**

*Pseudomonas aeruginosa*	10 (23%)
*Klebsiella pneumoniae*	6 (13%)
*Mycobacterium tuberculosis*	6 (13%)
*Enterobacter *sp.	5 (11%)
*Staphylococcus aureus*	5 (11%)
*Staphylococcus *negative-coagulase	4 (9%)
*Escherichia coli*	3 (7%)
*Acinetobacter calcoaceticus*	3 (7%)
*Serratia marcescens*	3 (7%)
Other	5 (11%)

### The 28-day and 6-month survival

**Figure 1:**
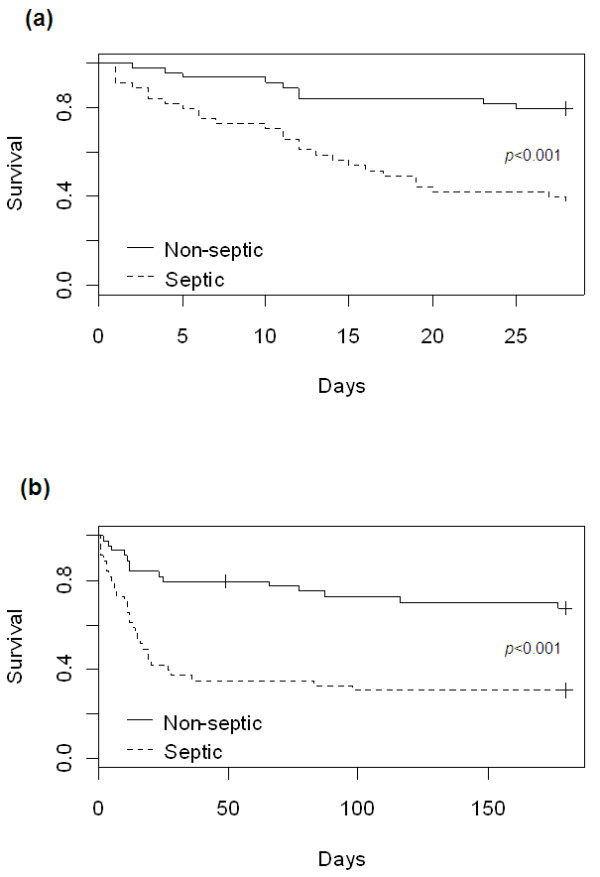
**Kaplan-Meier plot of survival up to 28 days (top) and 6 months (bottom) of sepsis and nonsepsis AIDS patients admitted to ICU**.

By using Cox proportional hazards regression, we estimated that the hazard of death at 28 days for sepsis patients was 4 times higher than that for nonsepsis patients (HR, 4.17; 95% CI, 1.96 to 8.90). Other factors associated with an increased hazard of death at 28 days were being in a hospital ward before entry to the ICU, and cardiovascular and respiratory dysfunctions (Table 4). The effect of sepsis on 28-day mortality was even stronger in the multivariate model, when controlling for confounding factors. The adjusted hazard of death at 28 days was almost threefold higher (HR, 3.13; 95% CI, 1.21 to 8.07) for sepsis patients compared with nonsepsis patients when controlling for age, time since AIDS diagnosis, HAART, location before ICU, and cardiovascular and respiratory dysfunctions. When considering 6-months of follow-up, sepsis also determined the highest hazard of death in both the crude (HR, 3.25; 95% CI, 1.75 to 6.16) and adjusted (HR, 3.35; 95% CI, 1.42 to 7.86) analysis (Table 4). The survival expectation of sepsis and nonsepsis patients was significantly different both at 28-day and 6-month follow-up time points (Figure 1, *P *values for log-rank and Peto tests <0.001) (Figure 1). No significant difference of 28-day or 6-month mortality was retrieved when on-admission or ICU-acquired severe sepsis was analyzed separately. When comparing follow-up periods, we noticed that, although the effect of sepsis was stronger during 28-day follow-up, it remained significant at 6 months.

**Table 4:** Crude and adjusted hazard ratios (HRs) and 95% confidence intervals for factors associated with 28-day and 6-month mortality, as estimated by using Cox proportional hazards regression

	28-Day mortality		6-Month mortality	
		
	Crude HR (95% CI)	Adjusted HR (95% CI)	Crude HR (95% CI)	Adjusted HR (95% CI)
Age (<40 years)	1.96 (0.99-3.89)		1.52 (0.84-2.77)	
Location before ICU (ward)	2.28^a^ (1.10-4.73)		1.93^a^ (1.02-3.64)	
Cardiovascular dysfunction	2.97^a^ (1.23-7.13)		2.12^a^ (1.05-4.30)	
Respiratory dysfunction	2.21^a^ (1.01-4.86)		1.58 (0.82-3.02)	
Severe sepsis/Septic shock	4.17^b^ (1.96-8.90)	3.13^b^ (1.21-8.07)	3.25^b^ (1.75-6.16)	3.35^b^ (1.42-7.86)
Time since AIDS diagnosis (<60 days)	0.96 (0.46-2.00)		0.95 (0.49-1.84)	
HAART	1.31 (0.68-2.52)		1.21 (0.67-2.18)	

AIDS, acquired immunodeficiency syndrome; ICU, Intensive Care Unit; HAART, highly active anti-retroviral therapy. Significant differences are shown as ^a^*P *< 0.05; ^b^*P *< 0.01. Adjusted HR includes adjustments for age, CD4 count, time since AIDS diagnosis, HAART, location before ICU, and cardiovascular and respiratory dysfunctions.

## Discussion

The improvement of HIV/AIDS management has led to changes in the observed clinical manifestations and outcomes of these patients in critical care units, with a decreasing trend in admissions due to opportunistic infections, whereas an opposing trend has been observed for other infectious and metabolic diseases [30]. Severe sepsis has emerged as a common cause of hospital admission for those living with HIV/AIDS [2,8,31]. In this prospective study, we demonstrated that severe sepsis is the main risk factor for hospital mortality in a cohort of HIV/AIDS critically ill patients.

Sepsis-related mortality was increased in both short- and longer-term follow-up. Indeed, sepsis patients had significantly higher in-hospital mortality than did nonsepsis patients. Severe sepsis was strongly associated with worse outcomes and was caused by bacterial infections, mainly of nosocomial origin. Nosocomial infections appear to be more common in patients with acquired AIDS compared with those not HIV infected [32]. Nosocomial infections have been associated with immunosuppression level, prior antibiotic use, and greater exposure to invasive devices such as intravenous catheters [33]. The CD4 cell count in our cohort was very low, and this could be associated with greater use of antibiotics for opportunistic infections prophylaxis or for treatment of bacterial infections contributing to a high rate of antibiotic resistance and, consequently, the development of nosocomial infection.

Pneumonia and bloodstream infections were the main sites of infections for almost all sepsis patients, and hospital-acquired bacteria composed the major part of the microbiology of severe infections. Lower-tract respiratory infection was also the main site of infection in two other studies [34,35]. Additionally, a high incidence of nosocomial bacteremia occurred in our cohort (43%). Bloodstream infections can negatively affect HIV/AIDS patients' outcomes [33,36-39]. In general, the microbiology of the HIV/AIDS sepsis patients was similar to that reported in non-HIV-infected patients. We observed a predominance of gram-negative and gram-positive bacteria, but also *Mycobacterium tuberculosis*, which was the main pathogen associated with severe sepsis in five of 44 patients. Tuberculosis, which is highly prevalent in developing countries, has been found to cause bacteremia in AIDS patients [36,40-44].

The development of organ dysfunctions was analyzed as a possible contribution to hospital mortality. In our study, cardiovascular and respiratory dysfunctions increased the hazards of death, especially in the short term. Respiratory dysfunction is closely associated to mechanical ventilation use and the presence of severe sepsis. Cardiovascular dysfunction could also be related to the presence of shock and the use of vasoactive agents, which are more frequent in sepsis patients. Use of mechanical ventilation [3,5,31,45,46], higher APACHE II or SAPS II scores [2,31,45,46], use of HAART during the ICU stay [45], and opportunistic infections [3,5,46] have been found to affect hospital mortality in a heterogeneous group of studies. Low CD4 cell counts, time since AIDS diagnosis, HAART treatment, or AIDS stage of disease, conversely, were not identified as risk factors for mortality in the post-HAART era [4,6,46].

In our cohort, almost 30% of patients had a recent AIDS diagnosis and were HAART naive, and an additional 16% of the patients were noncompliant HAART users. We found that these groups, who correspond to almost 50% of the patients in our study, were not subject to higher hospital mortality. Recent data has shown a greater impact of ICU care rather than HIV/AIDS management as major risk factors for death. Ventilatory management with lower tidal volumes was shown to be protective against mortality due to acute lung injury in critically ill HIV patients [47].

The main contribution of our study relies on the fact that sepsis is the most important risk factor for mortality in patients with HIV/AIDS admitted to the ICU. Other studies have not evaluated the diagnosis of severe sepsis or septic shock as an independent variable on survival analysis, whereas they gave greater emphasis to the presence of opportunistic infections or immunosuppression surrogates (CD4 cell count or viral load). Additionally, it provides new information on the microbiology of sepsis of the HIV/AIDS population, highlighting the occurrence of nosocomial severe infections and the great impact of sepsis on 1- and 6-month survival; second, confirming that CD4 the T-cell count, the HIV RNA level, and other HIV/AIDS variables were not predictive of 30-day and 6-month outcomes.

Otherwise, our study has limitations. The study was conducted in a single center specializing in HIV/AIDS patients care, lessening a greater generalizability; and a multicenter study is needed to confirm our findings regarding the effects of severe sepsis on HIV/AIDS critically ill patients. Although no prospective comparison was made with a cohort without HIV, similarities exist in the microbiology of infections and incidence of organ dysfunctions with the non-HIV/AIDS population reported in the literature [20,48,49]. Future studies are necessary to evaluate the long-term effects of sepsis on quality of life and on functional capacity.

Finally, although international efforts sought to reduce sepsis mortality, increased survival has not been observed in low- and middle-income countries (LMICs) [18,50-53]. New strategies to reduce the impact of sepsis, especially in LMICs, are timely. Those strategies should be able to deal with specific subgroups of patients, including the HIV/AIDS population. In this setting, the prevention of both nosocomial and community-acquired infections should be incorporated into sepsis guidelines as a cost-effective measure for LMICs.

## Conclusions

We demonstrated that severe sepsis is the main risk factor for hospital mortality in our cohort, significantly affecting short- and longer-term survival of HIV/AIDS critically ill patients. Mortality was shown to be more dependent on critically illness factors such as the presence of sepsis and the severity of organ dysfunction than on the HIV/AIDS-related characteristics, such as the level of immunodeficiency, use of HAART, or time since AIDS diagnosis. A high level of suspicion related to sepsis diagnosis coupled with initiatives related to nosocomial infection and sepsis prevention could contribute to decrease mortality in critically ill HIV/AIDS patients.

## Key messages

• Sepsis is the most important risk factor for mortality in HIV/AIDS patients admitted to ICUs, affecting short- and longer-term survival. HIV/AIDS variables, such as CD4 T-cell count and HIV RNA level, play a secondary role for the prognosis of critically ill HIV/AIDS patients.

• A high level of suspicion related to sepsis diagnosis coupled with initiatives related to nosocomial infection and sepsis prevention could contribute to decrease mortality in critically ill HIV/AIDS patients.

## Abbreviations

ACCP/SCCM: American College of Chest Physicians/Society of Critical Care Medicine; APACHE: Acute Physiology and Chronic Health Evaluation Score; CD4: cluster of differentiation 4; HAART: Highly Active Antiretroviral Therapy; HIV/AIDS: human immunodeficiency virus/acquired immunodeficiency syndrome; ICU: intensive care unit; NNRTI: nonnucleoside reverse transcriptase inhibitor; NRTI: nucleoside reverse transcriptase inhibitor; PI: protease inhibitor; PS: performance status; SAPS: Simplified Acute Physiology Score; SOFA: Sequential Organ Failure Assessment.

## Competing interests

The authors declare that they have no competing interests.

## Authors' contributions

All authors made substantial contribution to the study design and methods. AMJ, DMM, and FAB conceived of the study. AMJ, RTA, ECM, and HBB collected clinical and microbiologic data. PML performed the data analysis. AMJ, PML, and FAB drafted the manuscript, and DMM, EPN, and BG critically revised it for important intellectual content. All authors read and approved the final version of the manuscript.
